# Polyneuropathy monitoring in Parkinson's disease patients treated with levodopa/carbidopa intestinal gel

**DOI:** 10.1002/brb3.2408

**Published:** 2021-11-10

**Authors:** K. Amande M. Pauls, Jussi Toppila, Maija Koivu, Johanna Eerola‐Rautio, Marianne Udd, Eero Pekkonen

**Affiliations:** ^1^ Department of Neurology, Helsinki University Hospital and Department of Clinical Neurosciences (Neurology) University of Helsinki Helsinki Finland; ^2^ BioMag Laboratory, HUS Medical Imaging Center University of Helsinki and Helsinki University Hospital Helsinki Finland; ^3^ Department of Clinical Neurophysiology HUS Medical Imaging Center, Helsinki University Hospital Helsinki Finland; ^4^ Department of Gastrointestinal Surgery Helsinki University Hospital Helsinki Finland

**Keywords:** levodopa infusion, LCIG, Parkinson's disease, polyneuropathy

## Abstract

**Objectives:**

Levodopa‐carbidopa‐intestinal‐gel (LCIG) infusion is an effective treatment for advanced PD with motor fluctuations. Polyneuropathy occurs as a complication in 10–15% of patients. We wanted to assess the frequency of polyneuropathy in Finnish advanced Parkinson's disease (PD) patients with continuous LCIG infusion, and the value of different clinical monitoring parameters during follow‐up.

**Materials and methods:**

Patient records of PD patients started on LCIG infusion at Helsinki University Hospital who received nerve conduction studies at baseline and 6 months after treatment initiation were reviewed for epidemiological information, mini mental state examination, baseline and 6 months’ UPRDS‐III, weight, body mass index, levodopa dose (LD), plasma homocysteine levels, folate, vitamin B6 and B12.

**Results:**

Out of 19 patients (*n* = 6 on B‐vitamin substitution), two (10.5%) developed new‐onset polyneuropathy after initiation of LCIG therapy (*n* = 0 with vitamin substitution). Neuropathy was associated with significant weight loss (BMI reduction > 1.5), but not with other monitoring parameters. Homocysteine rose significantly in patients not substituted with B‐vitamin complex, but not in patients with B‐vitamin substitution. Homocysteine changes correlated with LD changes in the absence of vitamin B substitution. After oral B‐vitamin substitution, both patients’ polyneuropathy remained electrophysiologically and clinically stable.

**Conclusions:**

Rates of polyneuropathy in Finnish PD patients with LCIG treatment are comparable to previous studies. Patients’ weight should be included in regular follow up monitoring and can be used for patient self‐monitoring. Vitamin B substitution appears to reduce coupling between levodopa dose and homocysteine and may be useful to prevent polyneuropathy related to LCIG.

## Abstract 2

In a retrospective survey of continuous levodopa gel infusion in Finnish patients, 2/19 patients developed new‐onset polyneuropathy after initiation of LCIG therapy. Neuropathy was associated with significant weight loss, and homocysteine rose significantly in patients without B‐vitamin complex substitution, but not in patients with B‐vitamin substitution. Homocysteine changes correlated with levodopa dose changes in patients not treated with vitamin B complex.

## 1 INTRODUCTION

Infusion of levodopa‐carbidopa intestinal gel (LCIG) is an effective treatment for patients with advanced Parkinson's disease (PD) suffering from motor fluctuations despite optimal oral levodopa therapy (Antonini et al., 2016; Nyholm et al., 2012; Olanow et al., 2014). Previous studies have demonstrated beneficial effects, with lessening of motor fluctuations and increases in ON time without troublesome dyskinesia, and reduced OFF time (Antonini et al., 2016; Fernandez et al., 2015; Olanow et al., 2014). Non‐motor symptoms also improved (Antonini et al., 2017; Fernandez et al., 2015; Juhász et al., 2017; Standaert et al., 2017), resulting in an overall improvement in quality of life (Antonini et al., 2017; Fernandez et al., 2015; Juhász et al., 2017).

With increasingly wide use of LCIG infusion, a considerable rate of new onset polyneuropathy has been reported. Cases range from acute onset polyneuropathy to subacute and chronic onset. PD patients have an increased rate of polyneuropathy compared to healthy age matched controls (Comi et al., 2014). This could be a feature of the disease itself, that is, caused by alpha synuclein deposition in the peripheral nervous system, or due to cumulative levodopa exposure during treatment, possibly in conjunction with nutritional factors such B‐vitamin deficiency, and high serum homocysteine level (see Comi et al., 2014; Müller et al., 2013, for a review and see also Ceravolo et al., 2013; Rajabally & Martey, 2011; Toth et al., 2010; Toth et al., 2008).

The rates reported for new onset polyneuropathy during LCIG infusion therapy range from 4.5% to 19.0% (Merola et al., 2016; Uncini et al., 2015; Zadikoff et al., 2020). A cross‐sectional study comparing PD patients on different therapeutic regimens (LCIG infusion vs. oral levodopa vs. other dopaminergic treatment) found that the prevalence of polyneuropathy was highest in the LCIG group, and higher in patients treated with levodopa compared with other dopaminergic therapy (Mancini et al., 2014). Patients with polyneuropathy had significantly higher daily levodopa intake, and LCIG infusion patients had significantly lower vitamin B12 and folate levels, and higher homocysteine levels than the other groups. Levodopa daily dose correlated with homocysteine levels. A recent post‐hoc analysis of phase III data and registry data showed that the occurrence of polyneuropathy is increased in LCIG infusion patients with LD exceeding 2000 mg per day (Zadikoff et al., 2020). Vitamin B1 and B12 supplementation stabilized or improved polyneuropathy symptoms (Merola et al., 2016), and there were no cases of acute or subacute neuropathy in a cohort of patients substituted with vitamin B complex from onset of LCIG therapy (Rispoli et al., 2017). However, 19% of patients developed chronic polyneuropathy despite vitamin supplementation (Rispoli et al., 2017).

Recognition of early LCIG‐associated polyneuropathy is important to avoid irreversible long‐term damage. Knowledge of predisposing factors and causes can help clinicians identify LCIG patients at risk of developing polyneuropathy. It has been previously suggested that regular electrophysiological assessment is necessary for clinical follow‐up of LCIG infusion patients (Merola et al., 2016). Thus, we wanted to assess the relevance of routine electroneuromyography (ENMG) assessment and other clinical parameters in identifying patients at risk of developing polyneuropathy. In 2017 and 2018, routine ENMG measurements were carried out on all PD patients started on LCIG at the Helsinki University Hospital, once at baseline and once 6 months after initiation of LCIG infusion. In addition, clinical, laboratory, and electrophysiological parameters were collected.

## 2 MATERIALS and METHODS

At Helsinki University Hospital, advanced PD patients under consideration for LCIG are assessed routinely on an inpatient basis at baseline before the decision for LCIG, and at 6 months via an outpatient follow‐up visit, after which their follow‐ups are transferred back to the referring hospital. Permission to carry out the study was obtained from the responsible institutional review board at Helsinki University Hospital, and the study was carried out in accordance with the Declaration of Helsinki. Patient records of PD patients started on LCIG therapy from January 2017 to December 2018 were reviewed. From their electronic and other archived patient records, we extracted epidemiological data including gender, age, disease duration, mini mental state examination, as well as baseline and 6 months’ UPRDS‐III, height and weight, medication, levels of plasma homocysteine, folate, vitamin B6 and B12, and information on ENMG studies. Patients had at least two nerve conduction studies, baseline and at 6 months. In addition, we calculated body mass index (BMI in kg/m2), levodopa daily dose (LD), and levodopa equivalent daily dose (LED), as well as absolute and relative % changes from baseline to 6 months in BMI, LD, and LED, and plasma homocysteine levels. Vitamin levels of folate, vitamin B12, and B6 were classified qualitatively as normal, elevated, or reduced.

### 2.1 Levodopa daily dose (LD) and levodopa equivalent daily (LED) dose

LD and LED were calculated using the following conversion factors: Levodopa delayed release 0.75, levodopa/carbidopa with entacapone 1.33, levodopa gel (Duodopa) 1.11, rasagiline 100, pramipexole 100, ropinirole 20, rotigotine 30, amantadine 1 (Tomlinson et al., 2010).

### 2.2 Homocysteine and vitamin level assessment

Plasma homocysteine was assessed in umol/L. During the follow‐up time, the assessment methods for folate, vitamin B6, and vitamin B12 were changed at the Helsinki University Hospital central laboratory (folate: erythrocyte folate to total serum folate, nmol/L; vitamin B12: total serum vitamin B12 to active vitamin B12 (holotranscobalamine), pmol/L; vitamin B6: plasma pyridoxal phosphate to blood pyridoxal phosphate, nmol/L). Thus, measurements obtained using different assessment methods were available for different patients and sometimes within the same patient. Hence, vitamin data are classified as normal, reduced, or elevated.

### 2.3 Electrophysiological assessment

All patients included in the study underwent at least two ENMG assessments (baseline and 6 months after treatment initiation), which were carried out by the same clinical neurophysiologist (J.T.). Sensory nerve action potential (SNAP) and conduction velocity were studied in ulnar, radial, and sural nerves. Motor compound action potential, distal latency, and conduction velocity were studied in median, deep peroneal, and tibial nerves. Electromyography (EMG) was studied from anterior tibial muscle and gastrocnemius muscle. Polyneuropathy was diagnosed if at least two nerves showed reductions in amplitude and/or conduction velocity and the changes were not explained by a common focal compression neuropathy (such as carpal tunnel syndrome, ulnar nerve compression at elbow, peroneal nerve at head of fibula), or by common radicular symptoms. More than 50% reduction in SNAP or CMAP amplitude during follow up, or reduction of amplitude or conduction velocity compared to lower limits of common reference values was considered as an abnormal result in the follow up ENMG, leading to the diagnosis of polyneuropathy. More than 30% reduction in SNAP or CMAP during follow up was defined as at risk of developing polyneuropathy. In EMG, appearance of motor unit loss (rarefication) or fibrillations suggested recent or ongoing motor axonal damage.

### 2.4 Statistics

Statistical analysis was carried out using SPSS version 25 (www.ibm.com). Normality of data distribution was tested using a Kolmogorov‐Smirnov test. A paired *t*‐test was used for normally distributed data, and a Wilcoxon signed‐rank test for paired samples for non‐normally distributed data and samples of <10 subjects. Comparing patients with and without neuropathy, a Mann‐Whitney U‐test was used. Correlation testing was carried out using the Pearson correlation coefficient for normally distributed data, and Spearman correlation coefficient for non‐normally distributed data and samples of <10 subjects.

## 3 RESULTS

Over the period from 2017 to 2018, 19 patients were started on LCIG and were followed up with at least 2 nerve conduction studies. Patients’ epidemiological data can be found in Table 1. 6/19 patients were substituted with B‐vitamins (TrioBe, Meda Pharma, 3 mg vitamin B6, 0.5 mg vitamin B12, and 0.8 mg folate) from the onset of LCIG therapy, either because of elevated homocysteine levels and/or low vitamin levels at baseline, or because they wanted to take food supplements by their own choice.

**TABLE 1:** Patient demographics

					**Baseline UPDRS III**			**LD (mg)**			**LED (mg)**		
**Pat. no**.	**Age (years)**	**Gender**	**Disease duration (years)**	**Baseline MMSE**	**OFF** *	**ON** **^#^**	**6 mo. UPDRS III ON** *	**Base**	**6 mo**.	**LD change (%)**	**Base**	**6 mo**.	**LED change (%)**
1	41	f	10	20	73	37 + 4	n.a.	800*	1083*	35%	800	1083	35%
2	58	m	12	20	53	n.a.	33	1200	1571	31%	1380	1751	27%
3	81	m	18	30	38	22 + 3	18 + 2	1564*	1787*	14%	1564	1787	14%
4	70	f	9	24	26	15 + 3	n.a.	574	1199*	109%*	754	1199	59%
5	55	f	7	18	48	22 + 3	28 + 2	982	1158	18%	1102	1278	16%
8	80	m	13	24	49	27 + 2	24	1397	2280	63%	1520	2403	58%
9	67	f	16	27	42	12 + 1	18	848	1431	69%	1163	1746	50%
10	68	f	18	26	50	13	18	1081	1433	33%	1396	1748	25%
14	69	m	8	27	58	54	n.a.	973*	1212*	25%	973	1212	25%
16	73	m	12	28	34	19	27	665	1238	86%	845	1418	68%
17	73	f	16	29	58	15 + 2	19 + 2	973	1205	24%	1213	1445	19%
18	75	f	14	n.a.	63	39 + 2	39	1000	932	−7%	1052	984	−6%
19	70	m	8	24	38	33	31	915	1502	64%	1072	1554	45%
6	64	f	7	27	35	20 + 3	30 + 1	1200	1805*	50%*	1352	1805	34%
7	76	f	11	23	55	33	33	1463*	2337*	60%	1463	2337	60%
11	75	m	10	30	35	21	24	950*	2136*	125%	950	2136	125%
12	75	f	6	29	64	27	31	1650	2123*	29%*	2110	2123	1%
13	66	m	11	30	39	15 + 2	7 + 1	1746*	1584*	−9%	1746	1584	−9%
15	64	m	25	22	61	29 + 3	37 + 2	1248*	1606*	29%	1248	1606	29%
**Mean (range)**	**68.4 (41–81)**	**10 f, 9 m**	**12.2 (6–25)**	**25.4 (18–30)**	**48.4 (26–73)**	**25.2 (12–54)**	**26.1 (7–39)**	**1117 (574–1746)**	**1559 (932−2337**	**44.6 (−9–125%)**	**1248 (754–2337)**	**1642 (984–2403)**	**35.4 (−9–125)**

Light shading indicates patients that were substituted with vitamin B6, B12, and folate at baseline (others were not). Dark shading indicates patients who developed a polyneuropathy after treatment initiation. LD – Levodopa dose, LED – Levodopa equivalent dose, MMSE – Mini mental state examination, UPDRS III – Unified Parkinson's disease rating scale part III (motor assessment).

^#^ Global dyskinesia score, range 0 (no dyskinesia) to 4 (severe dyskinesia), given if available.

* Levodopa monotherapy.

**Change to levodopa monotherapy during observation period.

LD and LED both rose significantly over the observation period by a mean of 44.5% (LD) and 35.4% (LED) (paired samples t‐test, LD: baseline 1117 mg, 6 months1559 mg, *t* = 5.9, *p* < .01, LED: baseline 1248 mg, 6 months 1642 mg, *t* = 5.2, *p* < .01). 7/19 patients were on levodopa monotherapy at baseline. 3 additional patients were switched to monotherapy during the observation period, hence 10/19 patients received levodopa monotherapy at 6 months follow‐up.

None of the patients showed clinical or electrophysiological signs of polyneuropathy at baseline. 2/19 patients (10.5%) developed new‐onset polyneuropathy (with both clinical and electrophysiological signs) after initiation of LCIG therapy. One patient (patient 2) developed new‐onset axonal sensory polyneuropathy, with a glove and stocking pattern of numbness in the limbs. The other patient (patient 16) developed demyelinating sensory‐motor polyneuropathy, with a stocking‐like numbness in the lower limbs and reduction in vibration perception. Neither of the patients had clinical signs of muscle weakness. Both patients had a relative change in vitamin B6: the first patient's vitamin B6 level which was normal at baseline was reduced at 6 months. The second patient's vitamin B6 was elevated at baseline and dropped to normal levels at 6 months. Vitamin B12 and folate were normal at all times for both patients. Neither of the patients took B‐vitamin substitution at baseline due to normal B‐vitamin and homocysteine plasma levels. Both patients received oral vitamin B supplementation after symptom onset, after which their symptoms remained clinically stable. ENMG results were also stable or slightly improved on control assessment 6 months later. LCIG therapy was not interrupted in either case.

None of the patients on B‐vitamin substitution from baseline on developed new‐onset polyneuropathy. Neuropathy was associated with significant relative weight loss (Mann‐Whitney U‐test for independent samples, *p* = 0.013). BMI decreased by 1.5 kg/m2 (−5.9%) and 1.7 kg/m2 (−7.6%) over 6 months in the two affected subjects. LD and homocysteine changes were not significantly different between groups (Mann Whitney test, LD% change: 0.49, homocysteine plasma level change: *p* = 0.35). There was a relative reduction in vitamin B6 levels compared to baseline in both polyneuropathy patients, whereas levels of folate and vitamin B12 were in the normal range both at baseline and at 6 months. Patients’ clinical details are shown in Table 2.

**TABLE 2:** Patient clinical details

**Pat. no**	**BMI (kg/m^2^)**			**Homocysteine (umol/L)**			**Folate**		**Vitamin B12**		**Vitamin B6**		
	**Base**	**6 mo**.	**% change**	**Base**	**6 mo**.	**% change**	**Base**	**6 mo**.	**Base**	**6 mo**.	**Base**	**6 mo**.	**New‐onset PNP**
1	19.4	21.0	7.8	11	16	45.5	n	n	n	n	n	red.	
2	26.2	24.7	−5.9	13	22	69.2	n	n	n	n	n	red.	Axonal
3	22.6	23.2	2.5	11	22	100.0	n	n	elev.	n	n	n	
4	22.0	21.4	−2.8	13	34	161.5	n	n	n	n	n	red.	
5	22.7	23.4	3.4	11	11	0.0	n	n	n	n	n	n	
8	23.6	24.1	2.1	18	25	38.9	n	n	n	elev.	n	n	
9	17.3	16.7	−3.2	12	18	50.0	n	n	n	n	n	red.	
10	24.3	23.1	−4.8	22	20	−9.1	n	n	elev.	elev.	n	n	
14	18.3	18.6	1.6	8	13	62.5	n	n	elev.	elev.	n	n	
16	22.8	21.1	−7.6	19	22	15.8	n	n	n	n	elev.	n	Demyel.
17	23.6	24.4	3.4	12	13	8.3	n	n	n	elev.	elev.	red.	
18	18.4	18.4	0.2	14	10	−28.6	n	n	n	elev.	red.	n	
19	29.1	28.9	−0.5	21	30	42.9	n	n	n	n	n	n	
6*	17.6	20.8	17.6	27	14	−48.1	n	elev.	n	n	red.	elev.	
7*	19.8	20.6	4.0	22	14	−36.4	n	elev.	elev.	elev.	red.	elev.	
11*	29.9	30.3	1.5	8	9	12.5	n	elev.	n	n	elev.	n	
12*	n.a.	n.a.	n.a.	18	14	−22.2	n	elev.	n	elev.	elev.	elev.	
13*	23.8	24.0	0.7	16	15	−6.3	n	n	n	elev.	n	elev.	
15*	20.8	22.1	6.1	42	16	−61.9	n	elev.	n	n	red.	n	
**Mean (range)**	**22.4 (17.3–29.9)**	**22.6 (16.7–30.3)**	**1.5 (−7.6–17.6)**	**16.7 (8.0–42)**	**17.8 (9.0–34)**	**20.8 (−62–162)**		**elev. *n* = 5**	**elev. *n* = 4**	**elev. *n* = 8**	**red. *n* = 4, elev. *n* = 4**	**red. *n* = 5, elev. *n* = 4**	***n* = 2**

Rows in light grey highlight patients with B‐vitamin substitution from baseline onward (marked with *), dark grey highlighting marks patients who developed polyneuropathy.

BMI, body mass index; PNP, polyneuropathy; n, normal; elev., elevated; red., reduced.

**FIGURE 1:**
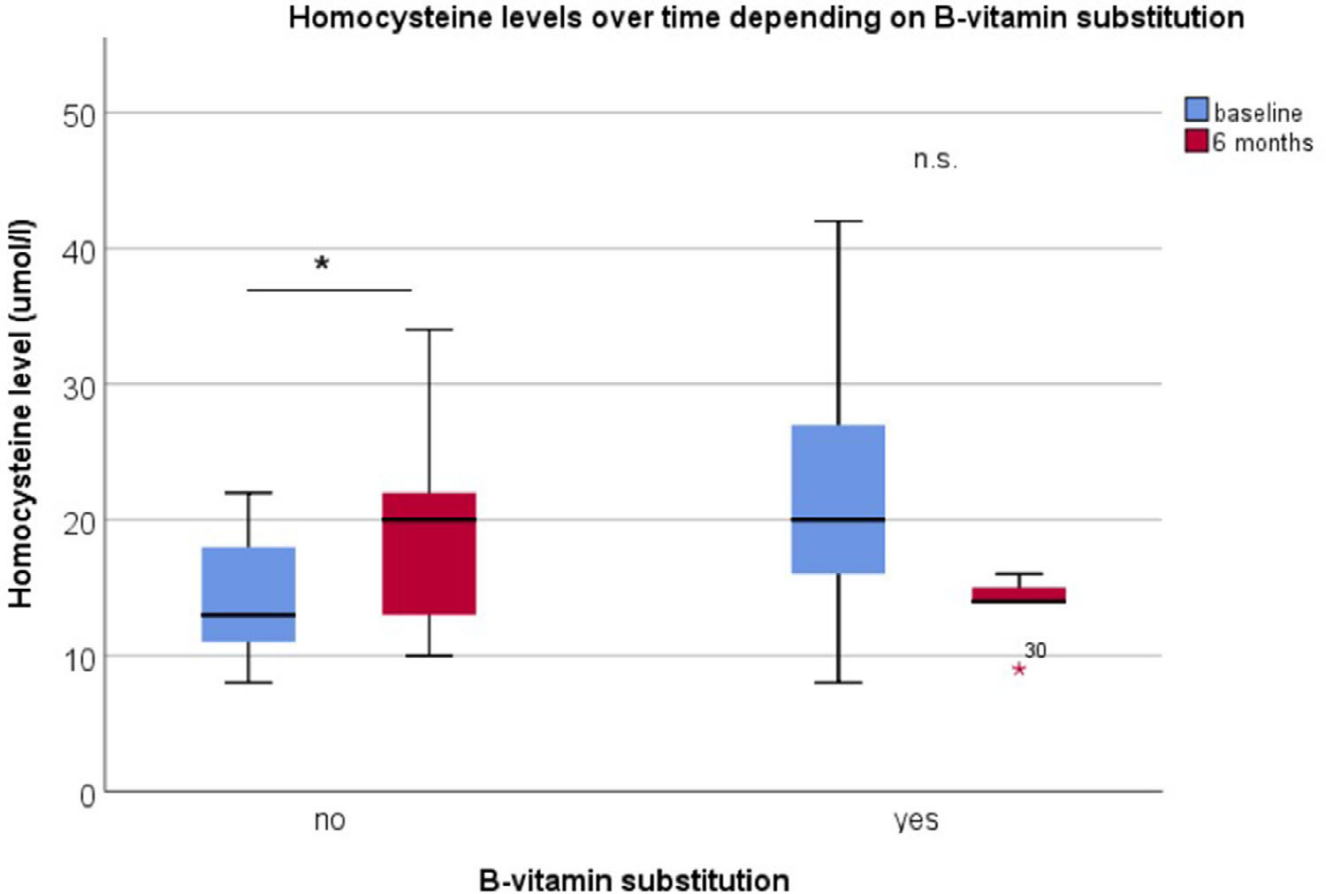
Homocysteine level change is influenced by B‐vitamin substitution. Homocysteine level increased in the no‐substitution group (14.2 to 19.7 umol/L, paired samples *t*‐test, *p* = 0.010), whereas homocysteine levels tend to decrease in patients with B‐vitamin substitution (22.2 to 13.7 umol/L, Wilcoxon signed‐rank test for paired samples, *p* = 0.058, n.s.)

**FIGURE 2:**
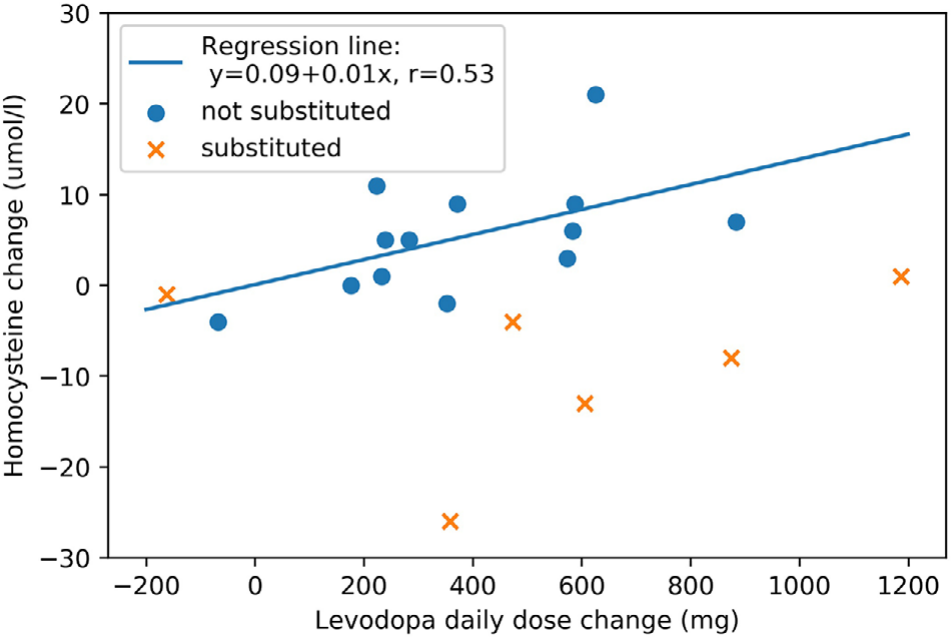
Increased homocysteine plasma correlated with daily LD increase in patients without vitamin B substitution (Pearson correlation, *r* = 0.64, *p* = 0.018), whereas there was no correlation in substituted patients (Spearman correlation, rho = 0.26, *p* = 0.62)

Homocysteine levels rose significantly from baseline to 6 months after LCIG initiation in patients without vitamin B complex substitution (14.2 to 19.7 umol/L, paired samples t‐test, *p* = 0.010), whereas there was non‐significant trend for decreases in homocysteine in patients with B‐vitamin complex substitution (22.2 to 13.7 umol/L, related‐samples Wilcoxon signed‐rank test, *p* = 0.058, n.s.), see Figure 1. Change in plasma homocysteine levels was positively correlated with change in LD (Pearson correlation, *r* = 0.64, *p* = 0.018) in patients without B‐vitamin substitution, see also Figure 2. In other words, increases in LD were accompanied by increases in plasma homocysteine levels in the absence of B‐vitamin substitution.

## 4 DISCUSSION

In summary, we observed a relatively low rate of new onset polyneuropathy after initiation of LCIG therapy (2/19 or 10.5%) in advanced PD patients who did not have any pre‐existing polyneuropathy. Both polyneuropathy cases occurred in patients who were not on B‐vitamin substitution at baseline, while none of the substitution patients developed polyneuropathy. Neuropathy was associated with significant relative weight loss (>5%, BMI change ≥ 1.5 kg/m2). Without B‐vitamin substitution, homocysteine levels rose significantly during LCIG therapy, whereas they tended to fall in patients with oral vitamin B supplementation. The rise in homocysteine levels was positively correlated with increased daily levodopa dose. Both new‐onset polyneuropathy patients received oral vitamin B supplementation after polyneuropathy diagnosis. LCIG therapy was not discontinued, and both patients' symptoms were stable 6 months after polyneuropathy onset, both clinically and on control ENMG examination.

### 4.1 Occurrence of polyneuropathy

The rates of polyneuropathy observed in our cohort are low compared to previous studies. None of our advanced PD patients had pre‐existing polyneuropathy, compared to 9–55% in previous studies (Merola et al., 2016; Rispoli et al., 2017; Toth et al., 2008). The incidence of new‐onset polyneuropathy (2/19, 10.5%) was relatively low, but in keeping with the reported range from 4.5% to 19.0% (Merola et al., 2016; Uncini et al., 2015; Zadikoff et al., 2020). Disease duration was shorter than in some previous studies (12.2 years on average), so cumulative exposure to levodopa was shorter, which may explain differences in baseline rates of polyneuropathy. Because of the relatively short follow‐up time of 6 months, chronic polyneuropathies were possibly missed. Further reasons for differences in both baseline occurrence of polyneuropathy and new‐onset polyneuropathy after LCIG initiation may include genetic differences (all patients were of Finnish descent), or dietary differences (Nordic Council of Ministers, 2014). It is known that the Finnish gene pool is quite distinct from other ethnicities (Lek et al., 2016), and previous studies on LCIG and neuropathy were carried out in several European countries and Australia (Zadikoff et al., 2020), Italy (Mancini et al., 2014; Merola et al., 2016; Rispoli et al., 2017), and Germany (Jugel et al., 2013). Epidemiological studies of the prevalence of polyneuropathy in Finland are unavailable but one study reports a rate of polyneuropathy of 1.4% in middle‐aged control subjects (Lehtinen et al., 1989).

### 4.2 LCIG infusion, weight loss, and polyneuropathy

We found that the occurrence of polyneuropathy was associated with significant weight loss over the first 6 months (BMI reduction ≥ 1.5 kg/m2). Weight loss in LCIG‐treated patients has been found to correlate with time spent with dyskinesia (Fabbri et al., 2019). Data on weight is only available in a few studies on LCIG treatment (Jugel et al., 2013; Klostermann et al., 2012; Zadikoff et al., 2020). The frequency of weight loss was higher in patients with LD ≥ 2000 mg daily (17.0% of patients), as was the rate of polyneuropathy, even though daily hours of dyskinesia were similar between the groups. However, an association between weight loss and polyneuropathy remains unclear (Zadikoff et al., 2020). Two of 20 PD patients treated with LCIG infusion developed acute sensorimotor axonal polyneuropathy with significant weight loss in both cases, as well as reduced vitamin B6 and folate and increased homocysteine (Klostermann et al., 2012). The number of electrophysiologically affected nerves was found to correlate with weight lost since initiation of LCIG infusion (Jugel et al., 2013).

### 4.3 Levodopa dose and homocysteine levels

The role of the so‐called one carbon pathway, which is relevant in levodopa metabolism, and its role in polyneuropathy are discussed in a review by Uncini et al. (2015). Homocysteine is a by‐product of levodopa metabolism, so high levels of daily levodopa dose raise plasma homocysteine levels, which has been shown before (Mancini et al., 2014; Zadikoff et al., 2020). Vitamin B6, vitamin B12, and tetrahydrofolate are involved in homocysteine metabolism, either by cleaving it into cysteine and methylmalonic acid (vitamin B6), removing it from the homocysteine cycle, or by restoring homocysteine to S‐adenosyl‐methionine (vitamin B12 and folate), the form relevant in metabolizing levodopa (Uncini et al., 2015). Thus, vitamin B complex supplementation reduces plasma homocysteine levels by increasing homocysteine metabolism, either via a pathway that removes it from the homocysteine cycle irreversibly (vitamin B6) or restoring it to the form active in the levodopa metabolism. If the availability of vitamins B6, B12, and folate is limited (as might be the case in PD patients with large daily levodopa doses), additional levodopa would increase homocysteine levels because homocysteine metabolism is running ‘at limit’. On the other hand, sufficient availability of the above vitamins as achieved by oral substitution would increase homocysteine metabolism, and hence reduce the amount of homocysteine produced per given amount of levodopa, reducing coupling between the two parameters. Administration of levodopa causes an increased metabolic demand for B‐vitamins B6, B12, and folate. Considerable weight loss associated with LCIG infusion suggests malabsorption of nutrients and vitamins from the gut, thus leading to relative lack of, for example, B‐vitamins or other nutrients relevant to the integrity of the peripheral nervous system.

### 4.4 Vitamin B levels, substitution, and polyneuropathy

Our polyneuropathy cases both showed a relative decrease in vitamin B6 levels compared to baseline but had normal vitamin B12 and folate at all times. Case studies have shown lowered vitamin B6, B12, and folate in individual patients with LCIG‐associated polyneuropathy (Klostermann et al., 2012). One study found reduced folate and vitamin B12 levels in PD patients on levodopa compared with other treatments, but there were no differences between patients with and patients without polyneuropathy (Mancini et al., 2014). Another study reports reduced vitamin B6 levels and elevated homocysteine, but normal folate and vitamin B12 levels, in patients with advanced PD with LCIG infusion or oral levodopa therapy (Loens et al., 2017). Almost all of the patients suffered from polyneuropathy, regardless of therapy.

On the other hand, none of the patients with vitamin B complex substitution developed polyneuropathy in our cohort, in keeping with previous studies (Rispoli et al., 2017). Vitamin B1 and B12 supplementation stabilized or improved polyneuropathy symptoms (Merola et al., 2016). Thus, vitamin B complex is relevant in the pathophysiology of LCIG‐associated neuropathy, but not reliable as sole clinical marker.

Is there something special about vitamin B6? As mentioned earlier, vitamins B6, B12, and folate are all required for levodopa metabolism and thus higher LD will deplete plasma levels. However, LCIG contains 20% carbidopa, which irreversibly binds to free vitamin B6 and deactivates vitamin B6‐dependent enzymes (Loens et al., 2017). Hence, the presence of carbidopa might further reduce vitamin B6 availability, deactivate B6‐dependent enzymes cleaving homocysteine and removing it from the homocysteine cycle, and thus, drive the development of levodopa‐associated neuropathy.’

### 4.5 Strengths and limitations

Patients were collected in one center only over a relatively short period of time (19 patients in 2 years), which means the collected data are quite homogeneous. Furthermore, all ENMG examinations were carried out by the same clinical neurophysiologist, eliminating inter‐rater variability of ENMG assessments. Limitations of the present study include the relatively small sample size and rather short follow‐up time of 6 months, which may reduce detection of chronic forms of polyneuropathy. Furthermore, laboratory procedures measuring plasma levels of B vitamins were changed during the study, limiting comparability and thus precluding correlation analysis.

## 5 CONCLUSIONS

Based on our findings, we suggest that upon initiation of LCIG, several monitoring measures should be collected, including patients’ weight, height, BMI, laboratory parameters (plasma homocysteine, vitamin B12, vitamin B6, folate). At baseline, patients should be also screened for clinical signs of polyneuropathy. During clinical follow‐up after LCIG initiation, the above monitoring measures should be repeated (e.g. at 6 months, 12 months, and yearly afterward), including examination for clinical signs of polyneuropathy. Patients should measure their weight regularly (at least once a month) and contact their treating center if weight loss exceeds 5% of baseline weight. ENMG should be carried out at low threshold especially in patients who develop significant weight loss (>5% from baseline weight over 6 months) during LCIG treatment. Patients should be made aware of the risk of polyneuropathy and its symptoms, as well as the possibility and relevance of (rapid) weight loss during LCIG treatment. Clinicians should be sensitized to the signs of polyneuropathy and possible predisposing factors, such as high or increasing level of homocysteine, significant weight loss, or low vitamin levels. The present results suggest routine initiation of oral vitamin B (B6, B12, and folate) supplementation upon LCIG initiation to prevent polyneuropathy.

## CONFLICT OF INTEREST

Amande Pauls has received lecture fees from Abbvie. Jussi Toppila has received lecture fees from UCB Pharma and is a consulting medical expert for SGS Fimko Ltd. Maija Koivu has received lecture fees from Abbvie. Johanna Eerola‐Rautio has received lecture fees from Abbvie, Nordicinfu Care AB and Allergan, and is a member of the advisory board of Nordicinfu Care AB. Marianne Udd has received lecture fees from Abbvie. Eero Pekkonen reports the following disclosures: Consulting fees by NordicInfu Care AB, Abbvie. Member of the advisory board: Abbvie. Lecturing fees: Abbott, Abbvie. Consulting neurologist for Finnish Patient Insurance Centre. Standing member of the MDS Non‐Motor Parkinson's Disease Study Group. Person Responsible of Trial in Finland: International DYSCOVER‐study (Dyskinesia Comparative Interventional Trial on Duodopa vs. oral medication) 2017–19, organized by Abbvie.

### TRANSPARENT PEER REVIEW

The transparent peer review history for this article is available at https://publons.com/publon/10.1002/brb3.2408

## Data Availability

The data that support the findings of this study are available from the corresponding author upon reasonable request and based on our institutional research permission protocol.
